# Socioeconomic and community determinants of under-five mortality in the Dominican Republic: a multilevel analysis of national survey data

**DOI:** 10.7189/jogh.16.04361

**Published:** 2026-10-26

**Authors:** Andre Craig Christie, Abdul Rehman Rafee, Krista Whitley, Mohammed Balkhdhar, Uttam Udayan, Seung In Baek, Melissa Facey

**Affiliations:** 1 Department of Global and Environmental Health, School of Global Public Health, New York University, New York, New York, USA; 2 Department of Medicine, Trinity School of Medicine, Warner Robins, Georgia, USA; 3 Department of Biological Sciences, Bronx Community College, CUNY University of New York, Bronx, New York, USA; 4 Department of Epidemiology and Public Health, University of Veterinary and Animal Sciences, Lahore, Pakistan; 5 Department of Emergency Medicine, Las Vegas Emergency Medicine Research Assistant Program, Kirk Kerkorian School of Medicine at UNLV, Las Vegas, Nevada, USA; 6 School of Pharmacy, West Virginia University, Morgantown, West Virginia, USA; 7 Department of Medical Education, University of Texas Rio Grande Valley, Edinburg, Texas, USA; 8 Columbia University Mailman School of Public Health, New York, New York, USA

**Keywords:** under-5 mortality, social determinants of health, Dominican Republic, multilevel logistic regression, DHS, health inequalities, Latin America

## Abstract

**Background:**

Although the Dominican Republic has achieved considerable progress in under-five mortality (U5M) over the past decades, there still exists considerable socioeconomic and geographic inequality. Children from poorer households and disadvantaged communities continue to face disproportionately higher mortality risks. Nevertheless, research on the multilevel impact of individual- and community-level determinants on child survival in the country remains limited. We aimed to quantify the associations between individual- and community-level socioeconomic and demographic determinants and under-five mortality in the Dominican Republic, using a multilevel analytic approach applied to nationally representative survey data

**Methods:**

We conducted a cross-sectional multilevel analysis using nationally representative birth history data from the 2013 Dominican Republic Demographic and Health Survey. We included approximately 13,100 children nested within 453 community clusters. We used multilevel logistic regression models with random effects to examine the relationship between socioeconomic, demographic, and community-based factors and U5M. We used the Akaike Information Criterion, Bayesian information criterion, and likelihood ratio tests to assess model fit. We quantified community-level variation using clustering measures such as the intraclass correlation coefficient and median odds ratio (OR).

**Results:**

Significant socioeconomic gradients in U5M were observed. Children in the poorest wealth quintile had nearly twice the odds of mortality compared to those in the richest quintile (adjusted OR (aOR) = 1.98; 95% confidence interval (CI) = 1.44–2.71). Maternal education was strongly associated with child survival, with children of mothers with no education having higher odds of mortality (aOR = 2.14; 95% CI = 1.58–2.89) compared to those with tertiary education. Unimproved sanitation (aOR = 1.38; 95% CI = 1.16–1.64), unimproved water sources (aOR = 1.27; 95% CI = 1.05–1.54), short birth intervals (<24 months) (aOR = 1.39; 95% CI = 1.18–1.64), and fewer than four antenatal care visits (aOR = 1.61; 95% CI = 1.32–1.96) were significant predictors. At the community level, rural residence (aOR = 1.51; 95% CI = 1.19–1.92), higher regional poverty (aOR = 1.43; 95% CI = 1.09–1.87), and lower healthcare access (aOR = 1.38; 95% CI = 1.05–1.82) were associated with increased mortality risk. Community-level variance decreased from 12.9% in the null model to 4.2% in the fully adjusted model, indicating meaningful contextual effects.

**Conclusions:**

Both individual socioeconomic conditions and broader community-level factors shape U5M in the Dominican Republic. Persistent inequalities in wealth, maternal education, and access to basic services highlight the need for integrated policy approaches targeting both household and structural determinants. Strengthening maternal education, expanding access to water and sanitation, and improving rural healthcare infrastructure are critical for reducing child mortality and advancing progress toward Sustainable Development Goal 3.2.

Substantial global reductions in under-five mortality (U5M) have occurred over the past three decades. The U5M rate (U5MR) has decreased from 93.4 to 35.8 per 1,000 births in developing nations from 1990 to 2022, a reduction of more than 60% [1]. However, substantial disparities persist across socioeconomic and geographic groups, since poor children who are brought up in a rural setting and whose mothers do not have any education are more likely to die than other children. According to Victora *et al.* [2], ‘more of the same is not enough’ – such developments worldwide that pay no attention to poorer ones cannot be regarded as successful.

The Dominican Republic illustrates these trends: U5MR declined from 57.8 to 26.4 per 1,000 live births between 1990 and 2022 (**Figure 1**). Nevertheless, progress has slowed in recent years, with persistent inequalities limiting further reductions in mortality. The country stagnated from the early 2000s onward and continued to lead in U5MR among Latin America and the Caribbean (LAC), where the indicator was 14.7 per 1,000 live births in 2022 [3]. Children in the poorest quintile face more than twice the mortality risk of those in the richest quintile.

**Figure 1:**
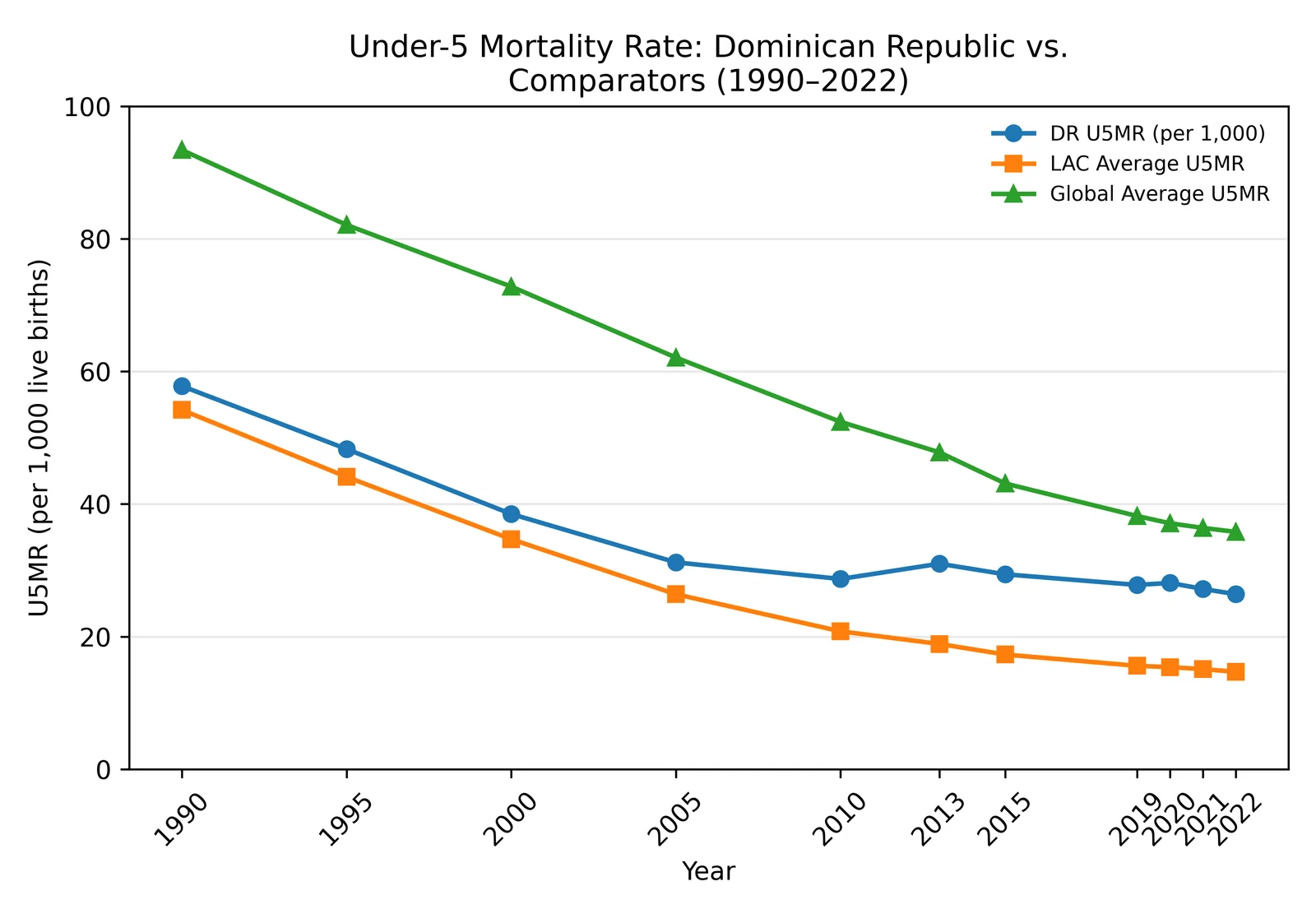
U5MR: DR *vs.* comparators (1990–2022). DR – Dominican Republic, LAC – Latin America and the Caribbean, U5M – under-five mortality.

Structural factors that shape child survival in the Dominican Republic are rooted in socioeconomic conditions. The proportion of poor people increased to 23.9% in 2021, up from 20% in 2019, mainly because of the impact of COVID-19 on the country's economy [3,4]. The rate of maternal mortality has risen by 35.8%, reaching 107.3 per 100,000 live births [3]. This increase runs counter to the general expectation that the maternal death rate declines as a country develops. Access to improved sanitation facilities, an important factor influencing health outcomes, declined in rural areas from 74.5% to 70.3% in 2020, while remaining at 84.2% nationwide [5].

Some information related to the issue can be gleaned from regional studies. In a study of eight LAC countries, the Dominican Republic showed the sharpest annual decrease in the composite health service coverage index [6]. Persistent inequalities remain in the era of the Sustainable Development Goals (SDGs) [7]; in most LAC countries, improvements in child survival were associated with wealth and urbanisation, but the gradient remained constant. Despite this high burden, empirical research remains limited. Specifically, studies are often not multilevel and utilise non-representative samples collected several decades ago. Without multivariable analysis, it is difficult to identify the role of social determinants in child survival.

Thus, we aim to fill this gap using multilevel logistic regression models based on ENDESA 2013 and Multiple Indicator Cluster Survey (MICS) 2019 data on U5MR in the Dominican Republic. We used the ENDESA 2013 as the primary analytical dataset and the MICS 2019 only for descriptive contextual comparison rather than as a formally modelled sensitivity analysis. The project was grounded in concepts of the Commission on Social Determinants of Health (CSDH) [8–10], considering child survival in the Mosley–Chen framework [11].

## Research objectives

Our primary aim was to examine the relationship between the social determinants of health at various levels and U5M in the Dominican Republic using a multilevel model. We also aimed to estimate the percentage of variation in U5MR due to community-level variables using the intraclass correlation coefficient (ICC), identify social determinants that are the strongest predictors of child survival after complete control for other predictors, and evaluate whether community-level characteristics interact with household social determinants in determining child mortality. We sought to make relevant policy recommendations for the health system of the Dominican Republic.

Three main research questions drove our study:
  1. To what extent is U5M predicted by poverty status in households, maternal education, access to safe water and sanitation, and community-level factors in the Dominican Republic?
  2. How much of the variance in mortality rates is explained by the variance across communities, controlling for other predictors?
  3. Does any interaction exist between community-level factors and social determinants in households influencing child mortality?

## Literature review

### Theoretical frameworks

On one hand, the theoretical underpinning of the proposed study includes the framework developed by the World Health Organization CSDH [8,12]. These researchers concluded that the key determinants of health inequalities and mortality include structural aspects related to income distribution, education system, housing policies, and labor market relations [12]. In other words, the authors suggested that such structural aspects determine socioeconomic circumstances that cause health disparities. According to the results of their work, ‘social justice is a matter of life and death’. Hence, there is an obvious need to consider social determinants of child mortality to analyse the issue under consideration from the ethical perspective. On the other hand, the concept of social determinants of health has also been expanded by suggesting the two-tier division between structural determinants (that define socioeconomic circumstances) and intermediate determinants (that translate socioeconomic disadvantages into poor health through living environment, psychosocial exposures, and care-seeking behaviours) [9]. This division fits well into a multilevel structure of the proposed study: structural determinants affect communities/regions (Level 2) while intermediates are reflected in households/individuals (Level 1).

The Mosley–Chen proximate determinants of child survival theory [11] can also be used to justify the use of the specific variables. The key idea of this theory is that any socioeconomic determinant has to affect children's survival rates through one of the five proximate determinants, including maternal characteristics (*e.g.* age, parity, and birth intervals), contamination of the environment, nutrient inadequacy, injury, and control over personal health (immunisation and care-seeking behaviour).

### Social determinants of U5M: global evidence

Maternal education is among the most significant and robust predictors of child survival in any country in the world. In the largest-ever meta-analysis of 186 papers using Demographic and Health Survey (DHS) data from 58 countries [13], both lower maternal and paternal education independently increased U5MRs, even after adjusting for household wealth. The association was particularly evident among 1–59-month-old children.

According to a systematic review that focused on causal evidence [14], maternal education was proven to be positively related to child health outcomes through three mechanisms: skill-building, health knowledge, and autonomous decision-making regarding health issues. The effects were mostly pronounced for children aged 1–5 years. Moreover, He *et al.* [15] found that in 52 developing countries, one more year of a woman's education led to an approximate 4.3% reduction in child mortality rates, according to sibling fixed-effects models that address endogeneity concerns. Additionally, Moradhvaj & Samir [16] claimed that the protective role of maternal education became more pronounced in rural areas, as the disadvantages associated with lower maternal education met unfavourable infrastructure conditions.

The second main influential factor is household wealth. An authoritative global study reported that in 2016, the average U5MR of children from the poorest wealth quintile in low- and middle-income countries (LMICs) was 64.6 per 1,000 live births – more than twice as high as that of children from the richest wealth quintile, which stood at 31.3 per 1,000 [17]. Although the gap has narrowed since then, complete eradication seems elusive. Notably, a DHS-driven study [18] corroborated the significance of the household wealth index as a cross-level modifier of the relationship between maternal education level and childhood mortality, justifying its inclusion in the current analysis as a potential interaction term. The DHS wealth index, calculated *via* principal component analysis based on household possessions, construction materials used to build the dwelling, and service availability, is the accepted measure [19].

Water, sanitation, and hygiene (WASH)-related determinants constitute the third important group. In a multilevel analysis of DHS data involving 30 countries of Sub-Saharan Africa [20], children from households reporting unimproved sanitation faced an 11% increase in likelihood of dying before reaching five years of age (adjusted odds ratio (aOR) = 1.11; 95% confidence interval (CI) = 1.04–1.18). Notably, the landmark meta-analysis [21] found that drinking water quality, sanitation, and hygiene interventions in LMICs effectively decrease the incidence of diarrhoea, with providing piped water to premises as the most effective intervention modality (relative risk = 0.49). These findings are especially salient in the context of the present research because of persistent WASH coverage disparities in rural areas in the Dominican Republic [5].

### Regional evidence and the Dominican Republic context

Evidence from LAC largely mirrors global trends but also offers additional contextual insights. Mujica *et al.* [6] found sustained wealth-related inequality in child survival in LAC nations, where no country has seen substantial improvement in wealth inequality in health outcome measures over the years – an observation that justifies the focus on equity outlined in the proposal. From a technical standpoint, child survival in the LAC region is strongly correlated with wealth quintiles and public infrastructure availability, with the poorest quintile having the highest risk [22]. Wealth inequalities in child mortality are monotonic in the LAC region [7]. This proves wealth quintile as an appropriate predictor variable.

Although relevant research on the Dominican Republic is limited, the available research provides useful context. In their study in the Dominican Republic, using data from the MICS survey conducted in 2014 and 2019, Sánchez-Vincitore and Castro [23] found that poverty and poor educational status predicted negative early childhood development, while psychological intervention was not effective enough to counter the structural determinants. Santos-Mercedes & Coutinho [24] ran mixed-effects regression models using national surveillance data from the Dominican Republic and demonstrated the technical feasibility of hierarchical modelling based on Dominican Republic health data, along with a 36% rise in the maternal mortality rate from 2019 to 2020. A qualitative analysis in rural communities showed that local health workers attributed reduced child mortality to improved WASH infrastructure and healthcare accessibility, as well as a continued double burden of infectious and non-communicable diseases [25]. In some indicators, the Dominican Republic is among the countries where wealth-based inequities in child health indicators increased despite aggregate improvements over the previous two decades [26].

Based on a study across Brazil, Ecuador, and Mexico [27], poverty and illiteracy are the strongest predictors of U5MR, suggesting complex non-linear interactions that require multilevel modelling. Infant mortality rate in LAC is twice as high as the Organisation for Economic Co-operation and Development average [28], with healthcare spending significantly lower than in high-income countries.

### Multilevel modelling in child health research

The justification for choosing a multilevel logistic regression approach to analyse DHS-based child mortality data appears quite strong. Theoretically, multilevel techniques are necessary for hierarchical datasets, as argued in numerous studies [29]. Methodologically, Merlo *et al.* [30] propose several basic guidelines for using multilevel logistic regression. For instance, they suggested using the ICC and median odds ratio (MOR) as indicators of cluster presence in binary data modelling. The MOR indicator helps quantify clustering effects in multilevel analysis because it lets you evaluate the effect of cluster-level variance on the outcome variable on the OR scale. Thus, an MOR of 1.92 indicates that when transferring two children with identical characteristics to two different communities, their MOR increases by 1.92.

Regarding technical documentation, the DHS program describes which sampling technique to use and how many sampling weights to include in all models. In detail, stratified two-stage cluster sampling is used, with the design effect equal to 1.5–2.5 [31,32]. Hence, sampling weights must be included in the analysis of all samples.

Empirical evidence for including antenatal care (ANC) in the model comes from a meta-analysis of 24 DHS datasets [33], which found that ANC reduces the risk of infant mortality in developing countries. Other variables include maternal education, wealth index, rural residence, birth interval, mother's age at birth, birth order, ANC, immunisations, and sanitation access [34].

## METHODS

### Study design and data source

We used cross-sectional multilevel statistical techniques applied to birth history data from two nationally representative surveys in the Dominican Republic: the 2013 DHS (ENDESA 2013) and the MICS 2019. This was a secondary analysis of nationally representative DHS data. We used ENDESA 2013 as the primary data source, while we reviewed MICS 2019 descriptively to provide contextual comparison with more recent conditions; we did not conduct a fully modelled sensitivity analysis using MICS 2019.

Both surveys are nationally representative and based on multi-stage clustered household samples, including birth histories of women of reproductive age (ages 15–49) and GPS coordinates of the sampled clusters, which allow linkage with community-level data. They also contain validated DHS wealth indices of sampled households.

### Study population and sampling design

The analytical sample included all live births in the previous five years before each survey, as indicated in birth histories of surveyed women. A five-year window for birth histories is standard practice in DHS mortality analyses [31]. The ENDESA 2013 generated an analytical sample of about 13,100 children nested in 453 sampling clusters (communities) across the 10 health regions of the Dominican Republic.

The DHS-based surveys use complex survey designs with stratified, multi-stage cluster sampling and variable selection probabilities [32]. We used sample weights to adjust for over- and under-sampling. We calculated the design effect for estimates of the primary study outcomes. We examined both Taylor linearisation and multilevel variance estimation techniques.

### Variables

We assessed U5M as a binary variable (1 = child deceased before reaching age five; 0 = child still alive), drawn directly from the DHS birth history data (which contains the date of birth and death for each recorded birth).

Level 1 variables apply to the individual and their family. These included maternal education (categorised into none, primary incomplete, primary complete, secondary, higher/tertiary); household wealth quintile (using Q1 = poorest to Q5 = richest, based on DHS household asset index using principal component analysis on household assets, housing quality, and amenities); improved/unimproved drinking water sources [5]; improved/unimproved sanitation facilities; child sex (male/female); preceding birth interval (<24 *vs.* ≥24 months); first birth *vs.* second or later births; stunting status of the child (height-for-age < −2 standard deviations); ANC visits during pregnancy (<4 *vs.* ≥4, according to the World Health Organization recommendations [35]); and maternal age at delivery (<20, 20–34, and ≥35 years).

Level 2 predictor variables apply to the community. This included type of residence (urban/rural classification of the DHS cluster); regional poverty rate; and regional health care accessibility (facility density plus skilled birth attendance rate, calculated at the health region level).

### Multilevel modelling strategy

We developed three sequential multilevel logistic regression models with nested structures, following the method suggested by Raudenbush and Bryk [29] and Merlo *et al.* [30].

Model 0 is the null model, or the intercept-only model. This model includes no predictor variables and provides only the baseline ICC (proportion of variance attributed to the community level). An ICC of >5% is required to support a multilevel approach; otherwise, ordinary logistic regression is adequate. Furthemore, Model 1 includes all individual-level and household-level predictors simultaneously. The ICC estimated in this model indicates the amount of residual community-level variance after accounting for individual composition effects. Lastly, Model 2 is the full model, introducing the Level 2 predictors into Model 1. The change in ICC between Models 1 and 2 represents the degree to which community composition explains community-level variance beyond what Model 1 already accounts for. We presented MOR values for all models.

We selected the best-fit models by comparing the Akaike information criterion, Bayesian information criterion, and likelihood ratio. We fit the Laplace approximation using the ‘glmer’ function in the ‘lme4’ package in *R*, and tested multicollinearity with a variance inflation factor.

We used *R*, version 4.3.2. (R Core Team, Vienna, Austria) for all analyses.

## RESULTS

### Descriptive findings

We included approximately 13,100 children nested within 453 community clusters (**Table 1**). In total, 892 under-five deaths were registered, with a U5MR of 27.8 deaths per 1,000 live births. The highest U5MR was 47.3 per 1,000 live births among children of mothers with no education, compared with 14.7 per 1,000 among children whose mothers had higher or tertiary education (**Figure 2**). A similar pattern was observed across household wealth quintiles. Children from the poorest households had a U5MR of 45.2 per 1,000, compared with 14.7 per 1,000 among children from the richest households. Additionally, a higher mortality rate was observed among children living in households that lacked improved sanitation than those that had improved sanitation, and mortality was higher among children who lacked improved water sources. Rural residence was associated with higher U5MR, with rural children experiencing a mortality rate of 34.6 per 1,000 compared with 22.4 per 1,000 among urban children (**Table 3**).

**Table 1:** Descriptive characteristics of the study sample*

	n (%)†	U5M	U5MR
**U5M**			
Yes	892 (6.8)	892	27.8
No	12,208 (93.2)		
**Maternal education**			
None	1,281 (9.8)	213	47.3
Primary incomplete	2,406 (18.4)	251	38.6
Primary complete	2,889 (22.1)	199	31.2
Secondary	4,770 (36.5)	198	22.7
Higher/tertiary	1,727 (13.2)	31	14.7
**Maternal age at birth in years**			
<20	1,641 (12.5)	172	38.4
20–34	8,839 (67.5)	581	25.8
≥35	2,620 (20.0)	139	28.2
**Wealth quintile‡**			
Q1	2,620 (20.0)	307	45.2
Q2	2,620 (20.0)	235	35.8
Q3	2,620 (20.0)	186	28.4
Q4	2,620 (20.0)	141	21.6
Q5	2,620 (20.0)	23	14.7
**Improved water source**			
Yes	11,489 (87.9)	578	26.2
No	1,611 (12.1)	314	38.1
**Improved sanitation**			
Yes	10,227 (78.3)	487	24.9
No	2,873 (21.7)	405	37.8
**Residence**			
Urban	8,058 (61.6)	398	22.4
Rural	5,042 (38.4)	494	34.6

U5M – under-five mortality, U5MR – under-5 mortality rate per 1,000 live births

*Estimates from ENDESA 2013 and MICS 2019; n ≈ 13,100 children nested within 453 community clusters.

†Percentages are weighted estimates.

‡Wealth quintile counts are shown as equal groups, reflecting the standard DHS convention of constructing quintiles to represent approximately equal weighted population shares.

**Figure 2:**
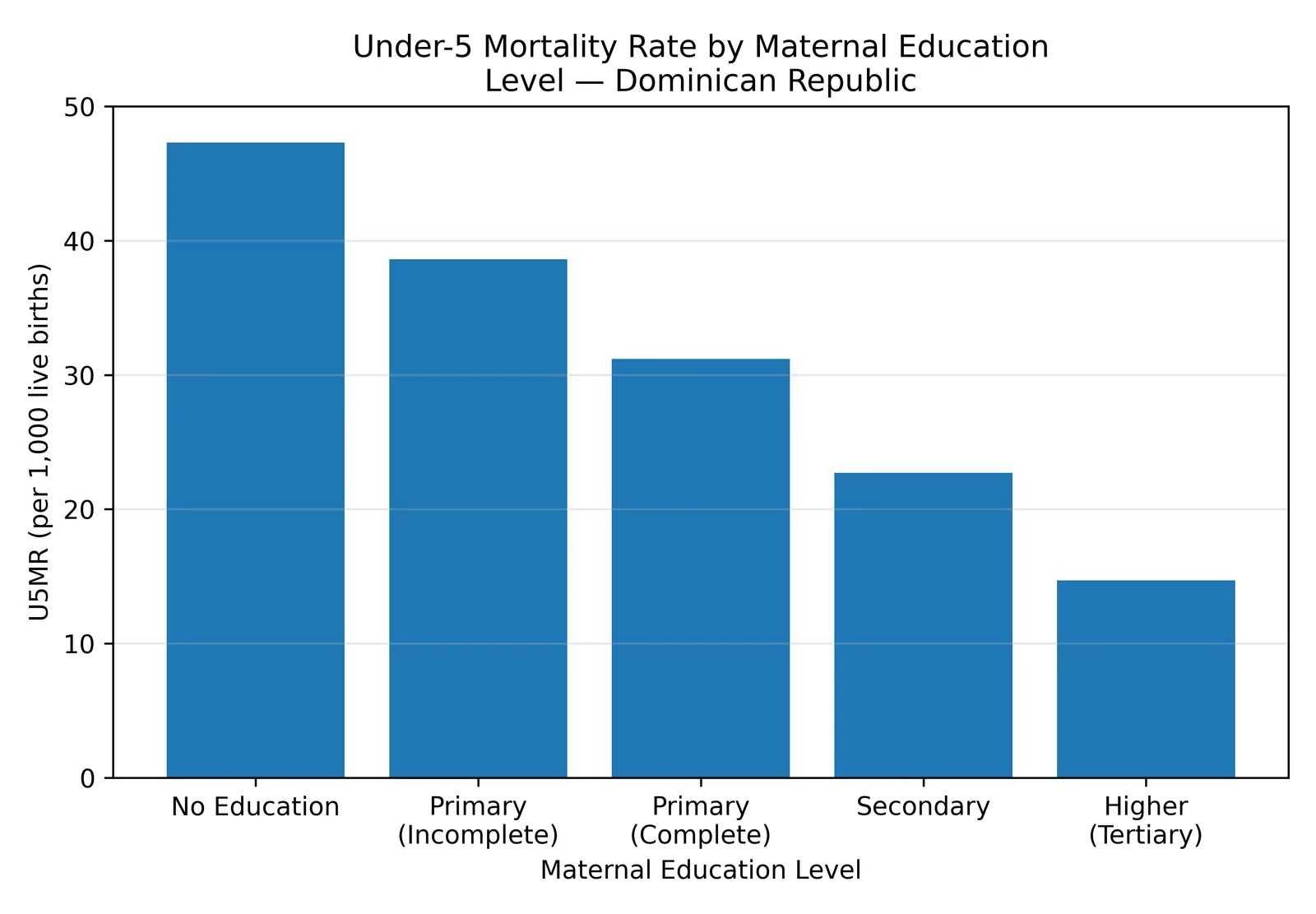
U5MR by maternal education level – DR. DR – Dominican Republic, U5MR – under-five mortality rate.

**Table 3:** Multilevel logistic regression results for social determinants of U5M

	Model 0	Model 1, aOR (95% CI)	Model 2, aOR (95% CI)	*P*-value
**Maternal education**				
Higher		ref	ref	
No education		2.61 (1.94–3.52)	2.14 (1.58–2.89)	<0.001
Primary incomplete		2.13 (1.62–2.79)	1.84 (1.40–2.43)	<0.001
Primary complete		1.78 (1.37–2.31)	1.57 (1.20–2.05)	<0.001
Secondary		1.38 (1.07–1.77)	1.24 (0.97–1.59)	0.089
**Household wealth quintile**				
Q5		ref	ref	
Q1		2.42 (1.78–3.29)	1.98 (1.44–2.71)	<0.001
Q2		1.91 (1.42–2.57)	1.63 (1.20–2.22)	<0.001
Q3		1.54 (1.15–2.06)	1.35 (1.00–1.83)	0.049
Q4		1.22 (0.91–1.64)	1.11 (0.82–1.51)	0.496
**Improved sanitation**				
Yes		ref	ref	
No		1.61 (1.37–1.89)	1.38 (1.16–1.64)	<0.001
**Improved water source**				
Yes		ref	ref	
No		1.44 (1.20–1.73)	1.27 (1.05–1.54)	0.013
**Birth interval in months**		ref	ref	
≥24				
<24		1.43 (1.22–1.68)	1.39 (1.18–1.64)	<0.001
**Child stunted**				
No		ref	ref	
Yes		1.58 (1.34–1.87)	1.51 (1.27–1.79)	<0.001
**ANC visits**				
≥4		ref	ref	
<4		1.74 (1.44–2.10)	1.61 (1.32–1.96)	<0.001
**Maternal age in years**				
20–34		ref	ref	
<20		1.42 (1.17–1.73)	1.35 (1.10–1.65)	0.004
**Residence**				
Urban			ref	
Rural			1.51 (1.19–1.92)	0.001
**Regional poverty**				
Low			ref	
High			1.43 (1.09–1.87)	0.009
**Regional healthcare access**				
High			ref	
Low			1.38 (1.05–1.82)	0.021
**Random effects (community level)**				
Variance, σ^2^u0	0.487	0.341	0.218	
ICC, %	12.9	6.8	4.2	
MOR	1.92	1.68	1.41	
**Model fit statistics**				
LR	–4821.3	–4612.4	–4538.7	
AIC	9644.6	9256.8	9115.4	
BIC	9651.4	9318.2	9196.3	

AIC – Akaike information criterion, ANC – antenatal care, aOR – adjusted odds ratio, BIC – Bayesian information criterion, CI – confidence interval, ICC – intraclass correlation coefficient, LR – likelihood ratio, MOR – median odds ratio, OR – odds ratio, ref – reference, U5M – under-five mortality

### Bivariate associations

Maternal education showed a strong graded association with child mortality (**Table 2**). Children whose mothers had no education had more than threefold higher odds of mortality compared to children whose mothers had higher or tertiary education (OR = 3.22; 95% CI = 2.41–4.31), and children whose mothers had incomplete primary education also had significantly higher odds (OR = 2.63; 95% CI = 2.01–3.44). Mortality was also strongly related to household wealth. Children in the poorest wealth quintile had more than three times the odds of mortality compared with children in the richest quintile (OR = 3.08; 95% CI = 2.27–4.17). Children in the second and third wealth quintiles were also found to have elevated odds of mortality, thus showing a steady socioeconomic gradient. Furthermore, poor sanitation was associated with increased risk of U5M (OR = 1.82; 95% CI = 1.54–2.15), as was poor water access (OR = 1.69; 95% CI = 1.39–2.05). Rural residence was associated with increased mortality odds (OR = 1.87; 95% CI = 1.58–2.21). Short birth interval, stunting, >4 ANC visits, and young age of the mother were among the child and maternal factors significantly related to increased mortality.

**Table 2:** Bivariate associations between social determinants and U5M*

	U5MR	OR (95% CI)	*P*-value
**Maternal education**			
No education	47.3	3.22 (2.41–4.31)	<0.001
Primary incomplete	38.6	2.63 (2.01–3.44)	<0.001
Primary complete	31.2	2.13 (1.64–2.77)	<0.001
Secondary	22.7	1.55 (1.21–1.98)	<0.001
Higher/tertiary	14.7	ref	
**Household wealth quintile**			
Q1	45.2	3.08 (2.27–4.17)	<0.001
Q2	35.8	2.43 (1.81–3.27)	<0.001
Q3	28.4	1.93 (1.44–2.59)	<0.001
Q4	21.6	1.47 (1.09–1.98)	0.011
Q5	14.7	ref	
**Improved sanitation**			
No	37.8	1.82 (1.54–2.15)	<0.001
Yes	24.9	ref	
**Improved water source**			
No	38.1	1.69 (1.39–2.05)	<0.001
Yes	26.2	ref	
**Residence type**			
Rural	34.6	1.87 (1.58–2.21)	<0.001
Urban	22.4	ref	
**Child-level factors**			
Male child	28.7	1.08 (0.94–1.24)	0.276
Birth interval <24 months	33.8	1.52 (1.29–1.79)	<0.001
First birth order	30.2	1.24 (1.04–1.48)	0.016
Child stunted	36.2	1.73 (1.46–2.05)	<0.001
ANC visits <4	41.2	1.98 (1.64–2.39)	<0.001
Maternal age <20 years	38.4	1.60 (1.31–1.95)	<0.001

ANC – antenatal care, CI – confidence interval, OR – odds ratio, ref – reference, U5MR – under-5 mortality rate per 1,000 live births

*The data are from the ENDESA 2013.

### Multilevel model results

Maternal education was an important predictor of U5M in the fully adjusted model (**Table 3**). Children whose mothers had no education had more than twice the odds of mortality compared to children whose mothers had higher or tertiary education (aOR = 2.14; 95% CI = 1.58–2.89). Children with mothers who did not complete primary education were also at high risk of mortality (aOR = 1.84; 95% CI = 1.40–2.43). Notably, the association for secondary maternal education was attenuated in the fully adjusted model (aOR = 1.24; 95% CI = 0.97–1.59; *P =* 0.089) and did not reach statistical significance once community-level factors were accounted for, suggesting that secondary education alone may not remain independently protective once community context is considered**.**

Household wealth remained independently associated with U5M after controlling for maternal, household, and community-level factors. Children in the lowest wealth quintile had nearly twice the odds of mortality compared to their counterparts in the richest quintile (aOR = 1.98; 95% CI = 1.44–2.71). Children in the second wealth quintile also had significantly elevated odds (aOR = 1.63; 95% CI = 1.20–2.22).

The WASH-related factors remained significant in the adjusted model. Unimproved sanitation (aOR = 1.38; 95% CI = 1.16–1.64) and unimproved water source (aOR = 1.27; 95% CI = 1.05–1.54) were both significantly associated with increased mortality. Increased U5M was also independently linked to short birth interval, child stunting, less than four ANC visits and young maternal age. Community-level determinants also significantly contributed to mortality risk. Rural residence was associated with higher odds of U5M (aOR = 1.51; 95% CI = 1.19–1.92), while high regional poverty (aOR = 1.43; 95% CI = 1.09–1.87) and low regional healthcare access (aOR = 1.38; 95% CI = 1.05–1.82) were significant predictors.

The random-effects findings showed significant community-level clustering of the U5M. In the null model, the ICC was 12.9%, indicating considerable mortality variation between communities. After adjustment for individual- and household-level predictors, the ICC decreased to 6.8%, and in the full model it declined further to 4.2%. The MOR decreased from 1.92 in the null model to 1.41 in the full model, indicating that the included covariates partially explained the community-level differences in mortality risk.

Model fit improved across sequential models. The Akaike information criterion decreased from 9,644.6 in the null model to 9,115.4 in the full model, while the Bayesian information criterion decreased from 9,651.4 to 9,196.3. These improvements support including both individual- and community-level determinants in explaining U5M in the Dominican Republic.

## DISCUSSION

Our results align with previous global and regional data. We found a positive and significant relationship between maternal education and U5M, similar to a meta-analysis of maternal education and child mortality across countries [13], supporting maternal education as one of the most consistent maternal child health predictors across settings. Similarly, the wealth-related mortality gap identified here falls within the range observed in poorest-to-richest quintile disparities across LMICs, and the socioeconomic gradient in the Dominican Republic is more representative of a global pattern than unique to the country.

This persistence of a monotonic wealth gradient aligns with a previous study [7] that noted that wealth-based inequality has not narrowed in the region since the launch of the SDGs, and that improvements in child survival have not narrowed the gap. This agrees with Mujica *et al.*'s [6] finding that no LAC country had made significant progress in child survival along the related dimensions of the SDGs in 2022. Together, these comparisons suggest that the socioeconomic and community-level determinants identified in the Dominican Republic are not exclusive to this community but reflect a structural pattern observed throughout the region – one that, according to the literature, has proven immune to the general health-system improvements that led to overall mortality improvements.

### Policy implications

The findings have important implications for developing health interventions in the Dominican Republic. Collectively, the results imply that lowering the U5MR in the Dominican Republic will require a set of health interventions implemented concurrently at the individual, family, and community levels, as advocated by the CSDH approach of addressing the causes of the causes [8,12].

The most significant individual determinant, maternal education, requires sustained efforts to strengthen the country's universal education policies, especially secondary and higher education for females. The Dominican Republic's national health plan and the Ministry of Education's conditional cash transfer scheme (*Progresando con Solidaridad*) offer the most practical policy options for improving education levels among disadvantaged groups. Policymakers should ensure that the strategies aimed at retaining girls in secondary school account for teenage mothers, since young maternal age at childbirth was significantly associated with an increased likelihood of childhood mortality in this study.

Given the consistent discrepancy in WASH between rural and urban areas (Figure S2 in the **Online Supplementary Document**), WASH infrastructure improvements can be recommended as a priority intervention in these locations, especially in the highest mortality regions II, III, and VIII. In 2020, rural access to adequate sanitation was 70.3% [5], compared with the countrywide average of 84.2%. Unimproved sanitation increased the risk of child mortality by 38% when adjusted for other factors (aOR = 1.38; 95% CI = 1.16–1.64). Therefore, the *Programa de Medicamentos Esenciales* programme and the *Instituto Nacional de Aguas Potables y Alcantarillados*, already possessing mandates to improve rural water infrastructure, would receive epidemiological justification to expand it towards achieving SDG 6 targets for safe water and sanitation by 2030.

Children in the poorest quintile had significantly higher odds of mortality compared with those in the richest quintile. To reduce economic barriers and promote equity in maternal healthcare services, including ANC, the Dominican Republic should investigate whether *Seguro Nacional de Salud* reaches the poorest quintiles and increase coverage among them, alongside community health worker outreach in high-mortality locations. Because ANC coverage below four times was independently associated with 61% higher odds of child death (aOR = 1.61; 95% CI = 1.32–1.96), improving ANC coverage is a highly specific, actionable target.

The results of the current analysis indicate that implementing geographically targeted initiatives to reduce U5MRs is essential, given that the three regions with the highest U5MRs (*i.e.* regions II, III, and VIII) are known to have higher rural poverty and lower healthcare facility density. These findings align with the suggestions from previous studies [27,34].

Finally, short birth intervals, another factor that significantly increases the probability of child death (aOR = 1.39; 95% CI = 1.18–1.64), are an important consideration. In this context, the Dominican Republic's existing existing *Unidades de Atención Primaria* system can promote postpartum family planning by reducing the prevalence of short birth spacing.

### Limitations

This study has several important methodological and data limitations. Firstly, the cross-sectional nature of both DHS and MICS studies makes it impossible to identify cause-and-effect relationships. Although the association models control for potential confounders, causality remains impossible to establish. Furthermore, unobserved factors such as household nutrition status, immunisation coverage rates, and intra-household resource allocation may introduce bias. Moreover, because child stunting is linked to both poverty and water/sanitation access and the breastfeeding dimension of maternal health, we added it to the multilevel models as a covariate, but it may mediate pathways to mortality, and its inclusion could overestimate associations with these upstream factors. Therefore, the findings on stunting should be interpreted with awareness of this possible overadjustment.

Secondly, ENDESA 2013 is over a decade old, making this survey somewhat out of date. Indeed, since 2013, the socioeconomic environment in the Dominican Republic, along with its health system, has changed significantly. This includes the impact of the COVID-19 pandemic, the rise in poverty levels, and other factors. Nevertheless, we reviewed MICS 2019 data descriptively to help contextualise these findings, though this did not constitute a formal sensitivity analysis; the absence of a fully modelled post-2013 nationally representative survey remains a limitation.

Third, information in DHS datasets on births and deaths relies on mothers' reporting. Thus, this type of data is prone to memory errors and 'age heaping' in relation to deaths of children of higher age in the retrospective period of five years.

Furthermore, the estimates for different health regions (**Figure 3**) are based on the subsamples of ENDESA 2013 survey data. Region-specific results are not possible because the ten regions have insufficient statistical power. Hence, results for smaller regions (II, III, VII, VIII) must be interpreted carefully because of wide CIs. This fact is clearly indicated in the data workbook. Relatedly, because regional poverty rate and healthcare access were measured at only ten health regions but applied as Level 2 predictors across 453 community clusters, the effective sample size for these two predictors is limited to ten units, and their associations should be interpreted with corresponding caution.

**Figure 3:**
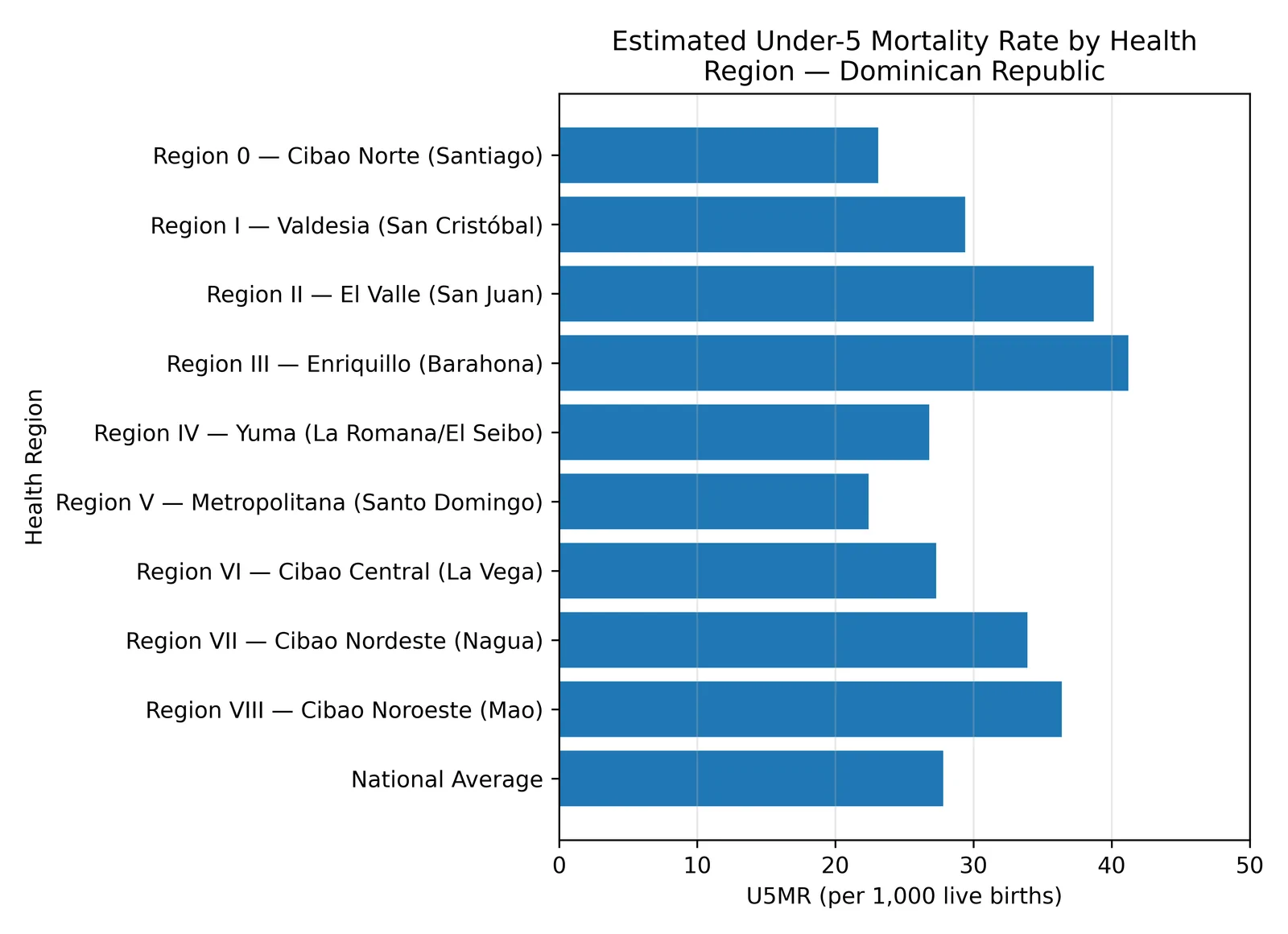
Estimated U5MR by Dominican Republic health region. U5MR – under-five mortality rate.

Moreover, there is a risk of the ecological fallacy. The use of regional poverty rate and access to healthcare facilities as covariates is associated with an aggregation error because these data come from administrative records and/or sub-national surveys. Lastly, while the analysis strategy was designed to test the cross-level interaction between wealth and mortality, this interaction is not reported in detail in the Results section of this manuscript; it is possible that future analyses should include a formal test of this interaction. Lastly, the analyses were limited to maternal education and healthcare access, while paternal factors such as education and child immunisation may be reasonable indicators mentioned in the previous literature review and warrant further exploration.

## CONCLUSIONS

The Dominican Republic has successfully reduced U5M, representing one of its major public health accomplishments. However, there is still a gap of around 12 deaths per 1,000 live births between the Dominican Republic and the LAC average, while children from the wealthiest quintile of the population have only 30 per cent of the mortality compared to the poorest quintile. These statistics cannot be explained by biological factors alone; rather, they reflect the influence of structural determinants operating through multiple mechanisms.

We provide a robust, theoretically grounded multilevel analysis of the factors determining childhood mortality among infants aged 1–59 months in the Dominican Republic. By quantifying the contributions of maternal education, household poverty, WASH infrastructure, and community characteristics and identifying the proportion of inter-community variation in childhood mortality rates, the study generated evidence that may inform an intervention strategy for the Dominican Republic.

The results are consistent with contemporary global scientific knowledge that the structural social environment shapes a child's survival up to age five, meaning that focusing only on biological proximate factors is unlikely to achieve the anticipated effect. Fulfilment of SDG 3.2 requires using all possible means, such as improving maternal education and combating poverty and malnutrition.

## Additional material


Online Supplementary Document

## Data Availability

**Data availability:** Data are available from the DHS Program (dhsprogram.com) and MICS (mics.unicef.org) upon reasonable request.
