# Supplementary material for: Socioeconomic and community determinants of under-five mortality in the Dominican Republic: a multilevel analysis of national survey data
Source: J Glob Health. 2026 Oct 26;16:04361. doi: 10.7189/jogh.16.04361 (PMC13706182; doi:10.7189/jogh.16.04361)

Supplement to: Christie AC, Rafee AR, Whitley K, Balkhdhar M, Udayan U, Baek SI, Facey M. Socioeconomic and community determinants of under-five mortality in the Dominican Republic: a multilevel analysis of national survey data. J Glob Health. 2026;16:04361.

Figure S1. Under-five mortality rate by household wealth quintile in the Dominican Republic compared with LAC and global LMIC averages

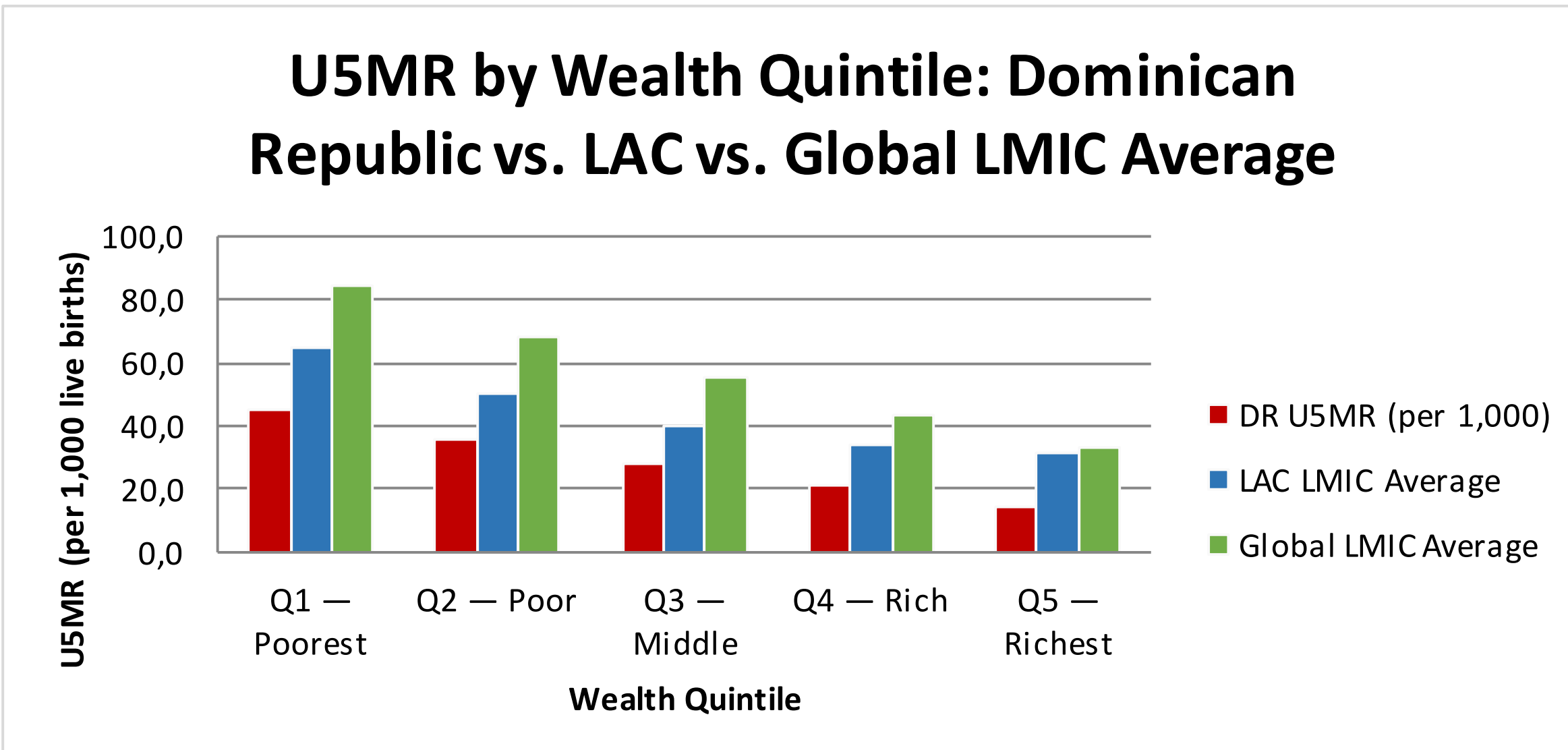

Figure S2. Trends in improved drinking water and improved sanitation coverage in the Dominican Republic, 2000–2020

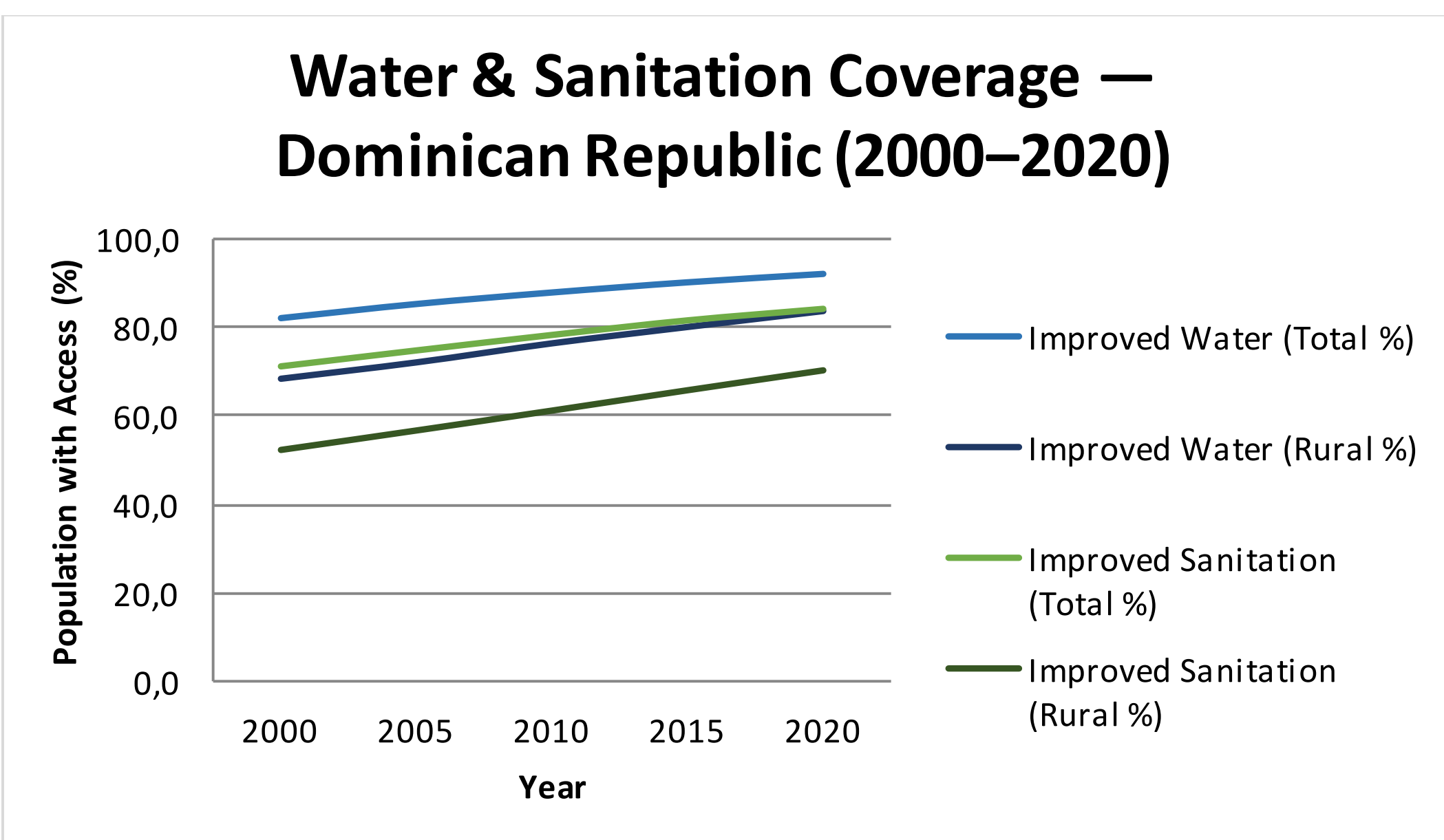

Supplement: Supplementary file 1 [file jogh-16-04361-s001.pdf]
